# Improved olfactory function following high-frequency transcutaneous auricular vagal nerve stimulation in post-COVID-19 patients with olfactory dysfunction

**DOI:** 10.1007/s00405-025-09701-5

**Published:** 2025-10-18

**Authors:** Susanne Weise, Mária Oravcová, Pauline Hanslik, Coralie Mignot, Yusuf Ozgur Cakmak, Thomas Hummel

**Affiliations:** 1 Smell & Taste Clinic, Department of Otorhinolaryngology, University Hospital Carl Gustav Carus, Technische Universität Dresden, Fetscherstrasse 74, 01099 Dresden, Sachsen Germany; 2 Point-of-Care Technologies Theme, Centre for Bioengineering and Nanotechnology, Dunedin, New Zealand; 3 Interventional Technologies Theme, Medical Technologies Centre of Research Excellence, Auckland, New Zealand; 4 Department of Anatomy, School of Biomedical Sciences, University of Otago, Otago, PO New Zealand

**Keywords:** Vagal nerve stimulation, Smell, Olfaction, Olfactory dysfunction, COVID-19

## Abstract

**Purpose:**

Previous studies in healthy individuals showed that high-frequency but not the low frequency non-invasive vagal nerve stimulation improves olfactory function in healthy individuals. The present study aimed to analyze the potential effects of non-invasive, high-frequency transcutaneous auricular vagal nerve stimulation (TA-VNS) on olfactory function in patients with olfactory dysfunction (OD) and healthy individuals.

**Methods:**

Patients with post-COVID-19 associated OD (*n* = 10) and normosmic individuals (*n* = 30) performed olfactory testing (Sniffin` Sticks), trigeminal testing (lateralization test), attentional tests (d2 test), ratings of odor intensity and pleasantness before and after receiving a TA-VNS for 10 min. Patients with OD received only TA-VNS while normosmic subjects underwent, in addition to TA-VNS, placebo stimulation (control condition) with electrode placement of TA-VNS, and active-control transcutaneous stimulation at the forearm in a randomized order with a minimum 24-h interval between sessions.

**Results:**

In patients with OD intensity ratings for fish and pleasantness ratings for eucalyptol increased and olfactory discrimination improved (*p* = 0.017) as opposed to olfactory threshold (*p* = 0.18). For both, patients with OD and healthy individuals results of the d2 attention test improved following the TA-VNS. In healthy individuals, there were no differences in olfactory discrimination nor threshold regarding the 3 stimulation modalities (TA-VNS, placebo (control condition), transcutaneous stimulation).

**Conclusion:**

These results seem to underline the functional connection of the olfactory system with the afferent vagus network. However, a ceiling effect for olfactory discrimination did not allow to observe a possible improvement in normosmic individuals. Further studies in patients with OD are needed to characterize this potential therapeutic effect of TA-VNS.

**Supplementary Information:**

The online version contains supplementary material available at 10.1007/s00405-025-09701-5.

## Introduction

Olfactory dysfunction (OD) affects around 20% of the population, with 5% being anosmic [1–3]. OD can have severe consequences, including increased risks for depression [4, 5], insecurity in social situations [6–8], higher risks for household accidents [9] and even an association with elevated five-year mortality rates [10].

Current therapeutic options consist mainly of olfactory training, surgery mainly for patients with OD due to chronic rhinosinusitis, and anti-inflammatory medication like corticosteroids or monoclonal antibodies against inflammatory mediators [3]. However, the olfactory function does not improve sufficiently for all patients, so further therapeutic options should be addressed [11]. These options include neuromodulation using the bottom-up functions of afferent cranial nerves [12]. The vagus nerve is the tenth cranial nerve, comprising both afferent and efferent fibers. It serves as the principal nerve of the parasympathetic system, regulating autonomous functions such as heart rate, digestion, respiration, and immune system. In recent years, vagus nerve stimulation (VNS) has emerged as a promising therapeutic approach for a broad range of medical conditions such as drug-resistant depression and some cases of refractory epilepsy [13, 14]. VNS can be delivered using invasive and non-invasive techniques. Invasive VNS requires the surgical implantation of a device. Non-invasive VNS external devices are used for transcutaneous vagal stimulation, commonly placed at the neck (transcutaneous cervical, TC-VNS) or ear (transcutaneous auricular, TA-VNS) [12]. TA-VNS delivers electrical stimulation to the auricular branch of the vagus nerve. It has been used as a therapeutic option in some cases of epilepsy, depression, and headaches [15]. Several studies had shown an improvement in cognitive functions in patients like attention, short-term memory, working memory, mood, and decision-making in patients [16–21]. Further, in healthy adults’ creativity and alertness, improved following non-invasive VNS [22, 23]. TA-VNS is generally well tolerated, with few side effects like skin irritation or redness [24].

In rats, it has been shown that VNS modulated electrical activity of the OB [25]. For high frequencies in VNS, an improvement in olfactory function was observed for normosmic and patients with OD [26], whereas no such improvement occurred with low frequencies in patients with epilepsy [27], depression [28] or Long-COVID [29]. However, despite the absence of significant changes in psychophysical olfactory function, an alteration in electrophysiological activity was observed in olfactory event-related potentials (ERPs) [27].

To examine potential changes in the olfactory function, patients with OD and healthy controls participated in identical measurements before and after TA-VNS. Further, to analyze the effect of the stimulation modalities and rule out the placebo effects healthy individuals underwent TA-VNS, placebo stimulation (control condition) with the electrode placement of TA-VNS, and another transcutaneous stimulation.

## Materials and methods

### Participants

The prospective study was conducted according to the Declaration of Helsinki at the Smell and Taste Clinic at the Department of Otolaryngology of the Technische Universität Dresden. It had been approved by the local ethics board (EK-400082021_1). All participants provided written informed consent and healthy participants received moderate financial compensation.

Healthy controls were recruited by flyers distributed at the University clinic and by word of mouth, while patients were referred by ENT specialists and general practitioners.

The inclusion criteria for patients were COVID-19-associated olfactory loss established through a Sniffin’ Sticks score (TDI) below 30.5 points [30]. For healthy controls, the inclusion criteria was a TDI score above 30.5 points. Further exclusion criteria for both groups were: age < 18 years, chronic rhinosinusitis (ruled out by a detailed medical history and a nasal endoscopy performed by an ENT specialist), systemic diseases associated with smell disorders like chronic renal failure, acute symptoms with allergic rhinitis, alcohol or drug abuse, pregnancy, epilepsy and individuals with implants such as pacemakers or other stimulators.

A total of 40 participants (aged 23–61 years, mean 32.0 ± 13.1 years, 24 women) were recruited for the study, consisting of 30 normosmic individuals as healthy controls and 10 patients with COVID-19-associated post-infectious OD. The gender distribution within the groups according to olfactory function can be found in Sup. Table 4.

### Procedure

**Fig. 1:**
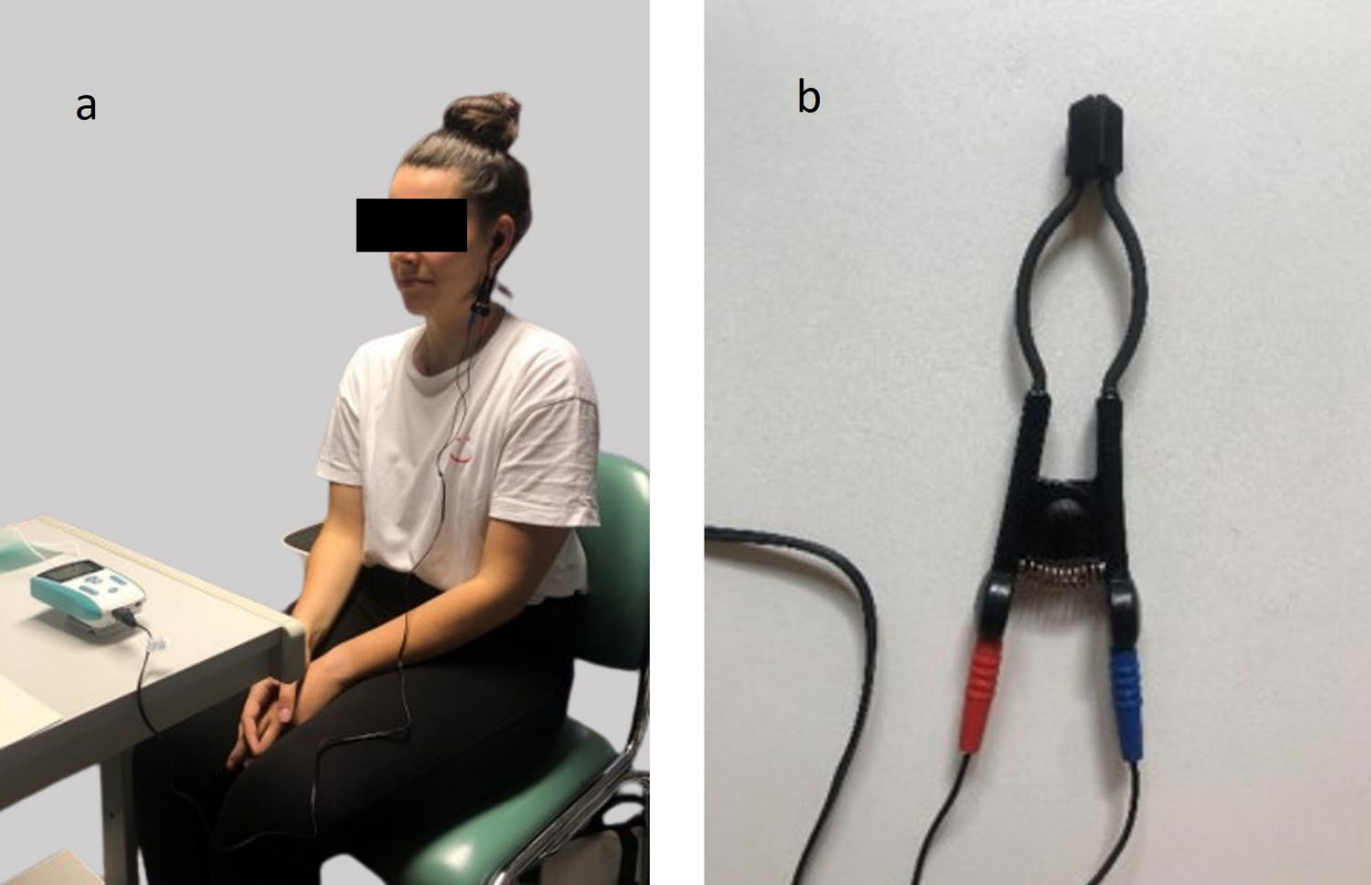
**a**) Location and setup of the non-invasive TA-VNS of the cavum conchae of the left ear, (paradigm as in Maharjan et al. 2018). **b**) auricular TENS electrode (Schwa-Medico, France)

Non-invasive TA-VNS was administered using transcutaneous electrical nerve stimulation (TENS) of the device “TENS ECO-2” (SCHWA-MEDICO, France) to the left ear (cavum conchae) according to the paradigm used by Maharjan et al. (2018) (see Fig. 1). This placement was chosen as it is considered safer and associated with fewer adverse events compared to stimulation on the right side [23]. Further, healthy individuals underwent additional transcutaneous active-control stimulation on the left forearm (same electrodes as in TA-VNS, electrode position ventral and dorsal forearm), and placebo stimulation (control condition) with no electrical stimulation but an attached electrode on the left ear to control for unspecific effects. Participants were informed that stimulations may not necessarily be perceived at all times, and that perception typically occurs only at higher stimulation intensities. The three stimulation modalities (TA-VNS, placebo (control condition), transcutaneous stimulation) in healthy individuals were applied in a randomized order for 10 min (see Figure for a schematic study paradigm) on three consecutive days with a 24-h test interval. To establish electrical conduction, water-based electrode gel (Dahlhausen®, Köln, Germany) was used between the skin and the clip. The stimulation parameters were set between 10 and 20 mA, pulse bandwidth 180 µs in square waveform, and 80 Hz over a stimulation period of 10 min. The stimulation was increased starting from 0 mA to a maximum of 20 mA and kept on a constant level so that the participants felt the stimulation as a tingling sensation. If the participants reported an unpleasant sensation or pain the stimulation was decreased so it was still bearable. The stimulation parameter was identical for TA-VNS and transcutaneous stimulation in healthy individuals.

Before the first test, all participants completed a detailed medical history including questions on conditions with a possible effect on olfactory function (e.g., nasal surgery, medication) and a questionnaire about the subjective importance of olfaction [31]. Further, olfactory testing, trigeminal function testing, the d2 test of attention, and ratings of odor intensity and pleasantness were conducted in a randomized order. Olfactory identification was assessed only before the first testing session. Following the stimulation, the olfactory threshold, discrimination, the d2 test of attention, trigeminal function, and ratings of intensity and pleasantness were measured in a randomized order (except for the olfactory threshold, which was tested first all).

The following provides a detailed description of the tests: the olfactory function was evaluated using the “Sniffin’ Sticks” (Burghart Messtechnik GmbH, Holm, Germany), including odor threshold, discrimination, and identification tests [32]. Based on this test, the olfactory function was categorized using the definitions of Hernandez et al. [33]: a score of 30.5 or higher indicated normosmia, between 16 and 30.5 indicated hyposmia, and fewer than 16 indicated anosmia [30, 34]. The intranasal chemical trigeminal sensitivity was tested using a lateralization test [35] using two squeezable bottles that were pressed simultaneously to deliver equal airstreams to the participants' nostrils, with only one containing an odorant (eucalyptol, C80601, Merck, Darmstadt, Germany; CAS number: 8000–48-4, 50% v/v diluted in 1,2-propanediol, SIGMA-ALDRICH, St. Louis, USA, CAS number: 57–55-6). Visually shielded subjects used a forced-choice paradigm to identify the nostril receiving the odor. Intranasal trigeminal function was classified as normal with a score of ≥ 15 out of 20 lateralization trials [36].

For the rating of odor intensity (0- “not intense at all” to 10 – “extremely intense”) and pleasantness (0- “extremely unpleasant” to 10 – “extremely pleasant”) the following odors were used: phenyl ethyl alcohol (PEA, rose-like, 20% v/v diluted in 1,2-propanediol, SIGMA-ALDRICH, St. Louis, USA, CAS number: 60–12-8), eucalyptol (1% v/v diluted in 1,2-propanediol) and fish sauce (20% v/v diluted in 1,2-propanediol, Squid, Thailand).

To assess concentration, as well as selective and sustained attention in tasks requiring focus, the d2 test of attention was used [37]. The test consists of rows of letters, where participants are instructed to quickly and accurately mark target symbols (the letter "d" with two dashes) while ignoring distractors. The analysis takes into account the error rate (F%) reflecting precision, concentration index (KL) and the number of processed target objects (BZO) referring to the processing speed.

**Fig. 2:**
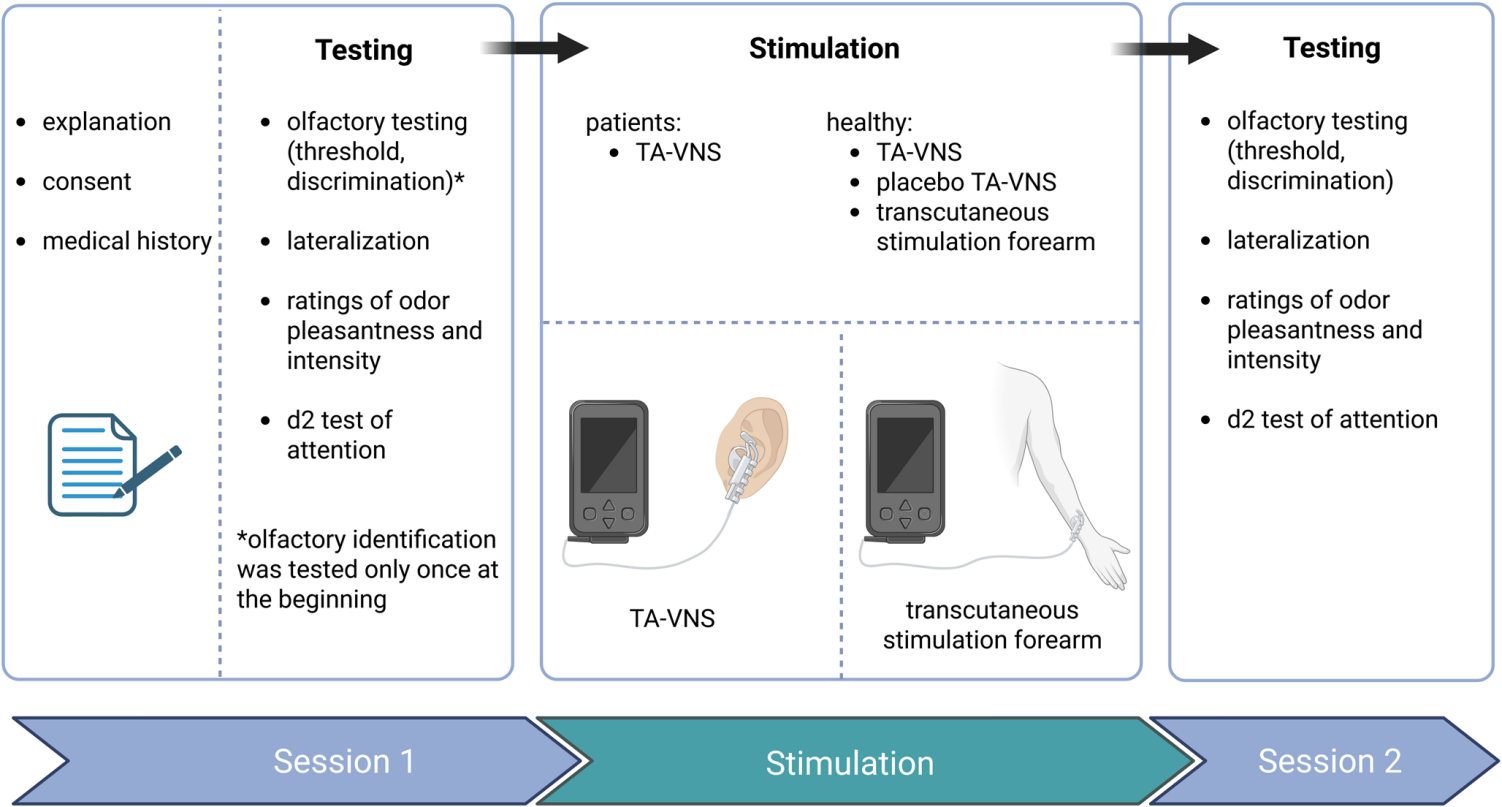
Schematic sequence of the study design: After a brief explanation of the study and obtaining informed consent, participants underwent olfactory testing for identification, discrimination, and threshold using Sniffin` Sticks [32], trigeminal testing using a lateralization test, and d2 test of attention in a randomized order. Following stimulation (for patients: TA-VNS; for healthy participants: TA-VNS, placebo stimulation (control condition) with electrode placement of TA-VNS, and transcutaneous stimulation at the forearm in a randomized order with a 24-h interval between each), olfactory discrimination, olfactory threshold, lateralization and d2 test of attention were reassessed. For healthy participants, an additional assessment of olfactory discrimination and olfactory threshold was conducted before each subsequent stimulation. Created in https://BioRender.com

All patients underwent nasal endoscopy by an ENT resident to rule out sinonasal causes of olfactory dysfunction, such as chronic rhinosinusitis with or without polyposis. Participants were instructed to avoid smoking or eating for 1 h before the testing session and to refrain from wearing strong fragrances on the day of testing (Fig. 2).

### Statistical analysis

Statistical analyses were performed using IBM SPSS Statistics (Statistical Packages for the Social Sciences, version 28.0; SPSS Inc., Chicago, Ill., USA). Descriptive statistics were obtained; continuous variables are expressed as means with standard deviation (SD), while categorical variables are presented as frequencies (percentages).

To compare healthy individuals and patients with OD, repeated-measures analysis of variance (rm-ANOVA) models were conducted for the following dependent variables (scores): olfactory threshold, olfactory discrimination, lateralization, intensity and pleasantness ratings of odors (PEA, Eucalyptol and fish sauce) and d2 test of attention. The measurement time point “session” (before vs after stimulation) was the within-subject variable and the group (patients with OD vs. healthy) was included as the between-subject variables. Results of the factor “group” were reported in supplementary. Rm-ANOVA was conducted for healthy individuals to compare the different stimulation sites, using “stimulation sites” TA-VNS, transcutaneous, placebo (control condition)) as the within-subject variable. Post hoc tests were conducted using pairwise comparison with Bonferroni correction. For olfactory discrimination and the d2 test of attention, the difference between sessions 2 and 1 was calculated. There were no outliers in the data for the dependent variables. The normal distribution for discrimination and identification was assessed using skewness and kurtosis, which were within the acceptable range according to Kim [38]. Pearson correlation was applied to assess the relationships between the variables od olfactory discrimination and the variables of the d2 test. A *p*-value of < 0.05 was considered statistically significant.

## Results

Patients were significantly older (Mean = 51.8 ± 12.1 years) than healthy individuals (Mean = 25.4 ± 2.5 years, t(9.26) = 6.84, *p* < 0.001 [17.71; 35.09]). There were more female participants in the patient’s group (90%) compared to healthy controls (50%, Fisher exact test: *p* = 0.032), see Sup Table 4.

### Olfactory and trigeminal function of the studied population

According to inclusion criteria, all healthy individuals were normosmic (mean TDI score = 38.9 ± 4.0; TDI score range 32.25 to 47.5). Patients with COVID-19-associated OD had a poorer olfactory function (mean TDI score = 20.5 ± 4.1; TDI score range 14.0 to 26.5; t(38) = 12.6, *p* < 0.001) compared to healthy individuals (see Sup Table 5, and Sup Table 6 for descriptive data), of whom 8 were hyposmic and 2 anosmic. For the olfactory function concerning gender distribution within the groups, see Sup Table 5. In healthy controls, the lateralization ability tended to be higher compared to patients (*p* = 0.058, t(38) = 1.95, 95%-CI[0.17, 3.84]). However, 80% of patients and 93% of healthy individuals exhibited normal trigeminal function according to the normative data provided by [36].

### Comparison of patients with OD vs. healthy controls

Following TA-VNS there was a significant effect for olfactory discrimination, d2 test of attention, and for some odors regarding their perceived pleasantness and intensity, see Table 1.

**Table 1:** *p*-values of the main effect session and the interaction effect between session and group. (*p*-value of < 0.05 was considered statistically significant and highlighted in bold)

	Main effect session	Interaction effect between session and group
Olfactory threshold	*p* = 0.18	*p* = 0.78
Olfactory discrimination	***p* = 0.017**	***p* = 0.031**
Lateralization	*p* = 0.38	*p* = 0.73
Intensity ratings Fish PEA Eucalyptol	***p* = 0.012** *p* = 0.051 *p* = 0.68	*p* = 0.14 *p* = 0.051 ***p* < 0.001**
Pleasantness ratings Fish PEA Eucalyptol	*p* = 0.13 *p* = 0.95 ***p* = 0.032**	*p* = 0.09 *p* = 0.051 ***p* = 0.007**
d2 test of attention, BZO KL F%	***p* < 0.001** ***p* < 0.001** ***p* < 0.001**	***p* < 0.001** ***p* < 0.001** ***p* < 0.001**

**Fig. 3:**
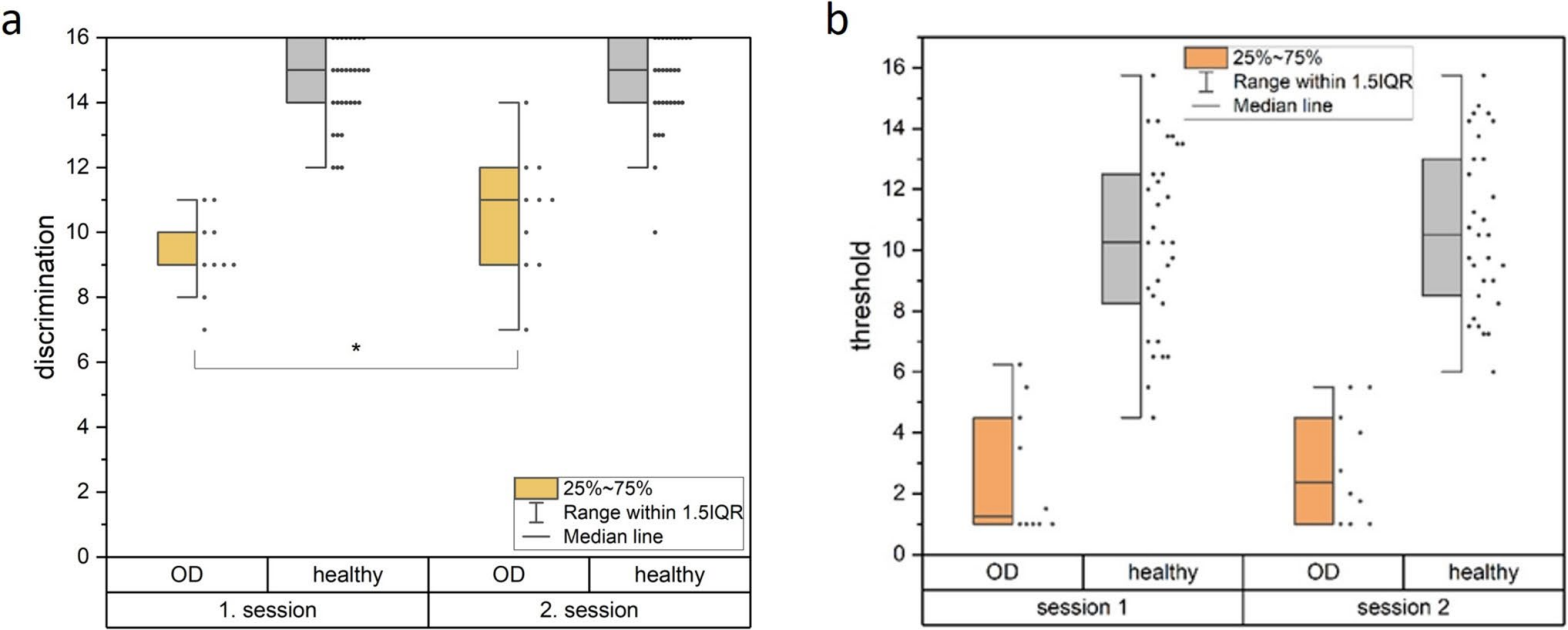
Olfactory discrimination (**a**) improved between the two sessions for patients with OD compared to healthy individuals (*p < 0.05) as opposed to no significant changes seen for olfactory threshold (**b**)

Regarding the olfactory discrimination, there was a significant main effect for the session (F(1, 38) = 6.19, *p* = 0.017) and the interaction of session and group (F(1, 38) = 5.70, *p* = 0.031). The pairwise comparison showed, that patients exhibited a significant improvement in olfactory discrimination (*p* = 0.009, *M*_Diff_ = 1.30, 95%-CI[0.34, 2.26]) as opposed to the healthy controls (*p* = 0.81, *M*_Diff_ = 0.07, 95%-CI[0.49, 0.62]), for the olfactory threshold, no change was observed between the two sessions in the OD and healthy groups. see Fig. 3

For the intensity ratings significant main effects for the session for the odor fish (F(1,38) = 7.05, *p* = 0.012), a trend for PEA (F(1,38) = 4.05, *p* = 0.051) and the interaction of session and group for the odor Eucalyptol (F(1,38) = 13.34, *p* < 0.001), a trend for PEA (F(1,38) = 4.05, *p* = 0.051) emerged. The pairwise comparison showed lower intensity ratings in the second session for healthy controls (PEA: *p* < 0.001, *M*_Diff_ = 0.73, 95%-CI[0.36, 1.10], Eucalyptol: *p* < 0.001, *M*_Diff_ = 1.13, 95%-CI[0.57, 1.70]), but not for patients (PEA *p* = 1.00, Eucalyptol *p* = 0.07). However, patients rated the intensity for the odor fish higher in the second session (*p* = 0.021, *M*_Diff_ = 1.10, 95%-CI[0.18, 2.02]) as opposed to the healthy controls (*p* = 0.26).

The pleasantness ratings for eucalyptol showed differences respectively the main effect for the session (F(1,38) = 4.92, *p* = 0.032) and the interaction of factors session and group (F(1,38) = 8.26, *p* = 0.007). The pairwise comparison showed that patients rated the pleasantness of Eucalyptol higher in the second session compared to the first one (*p* = 0.006, *M*_Diff_ = 1.30, 95%-CI[0.41, 2.20]), however, there was no such difference for healthy controls (*p* = 0.52).

**Fig. 4:**
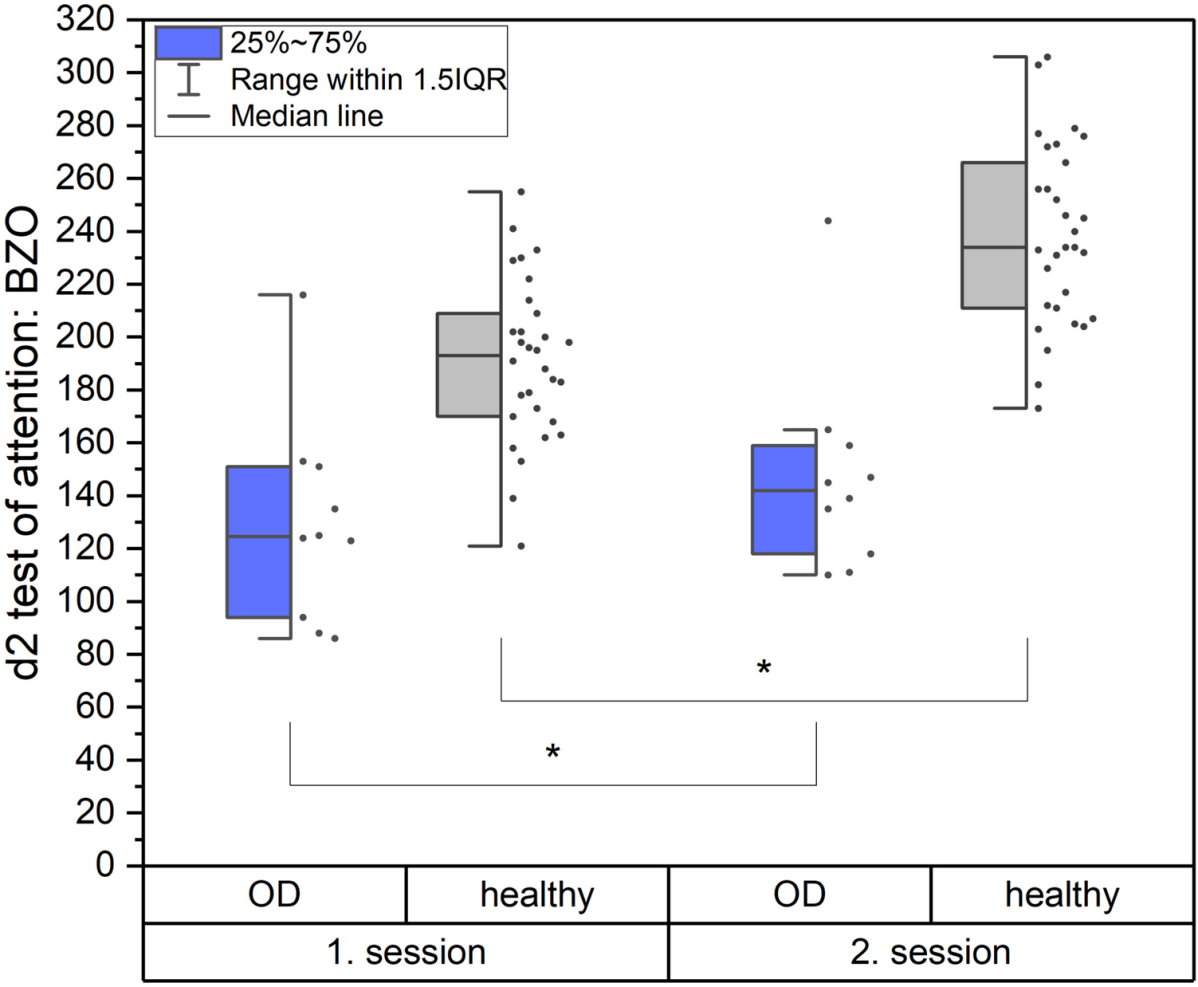
Fig. 4 Improvement from session 1 to session 2 in regarding processed target objects (BZO) in the d2 test of attention for both OD patients and healthy individuals. * *p* > 0.05

For the “d2 test of attention”, a significant main effect was observed for session (BZO (F(1,38) = 124.10, *p* < 0.001) and F% (F(1,38) = 55.95, *p* < 0.001) and interaction of session and group (BZO (F(1,38) = 25.26, *p* < 0.001) and F% (F(1,38) = 27.71, *p* < 0.001), see Sup Table 7 for descriptive data. The pairwise comparison showed that both patients and healthy controls improved from session 1 to session 2 regarding BZO (patients *p* = 0.001, *M*_Diff_ = 17.8, 95%-CI[7.5, 28.0]; healthy controls *p* < 0.001, *M*_Diff_ = 47.1, 95%-CI[41.2, 53.0]) and that healthy controls improved for KL (*p* < 0.001, *M*_Diff_ = 89.5, 95%[74.7, 104.4]) and F%: (*p* < 0.001, *M*_Diff_ = 33.14, 95%-CI[27.87, 38.40]). However, patients did not improve significantly regarding KL (*p* = 0.43) or F% (*p* = 0.21) (Fig. 4).

### Relation of attention and olfactory discrimination

**Fig. 5:**
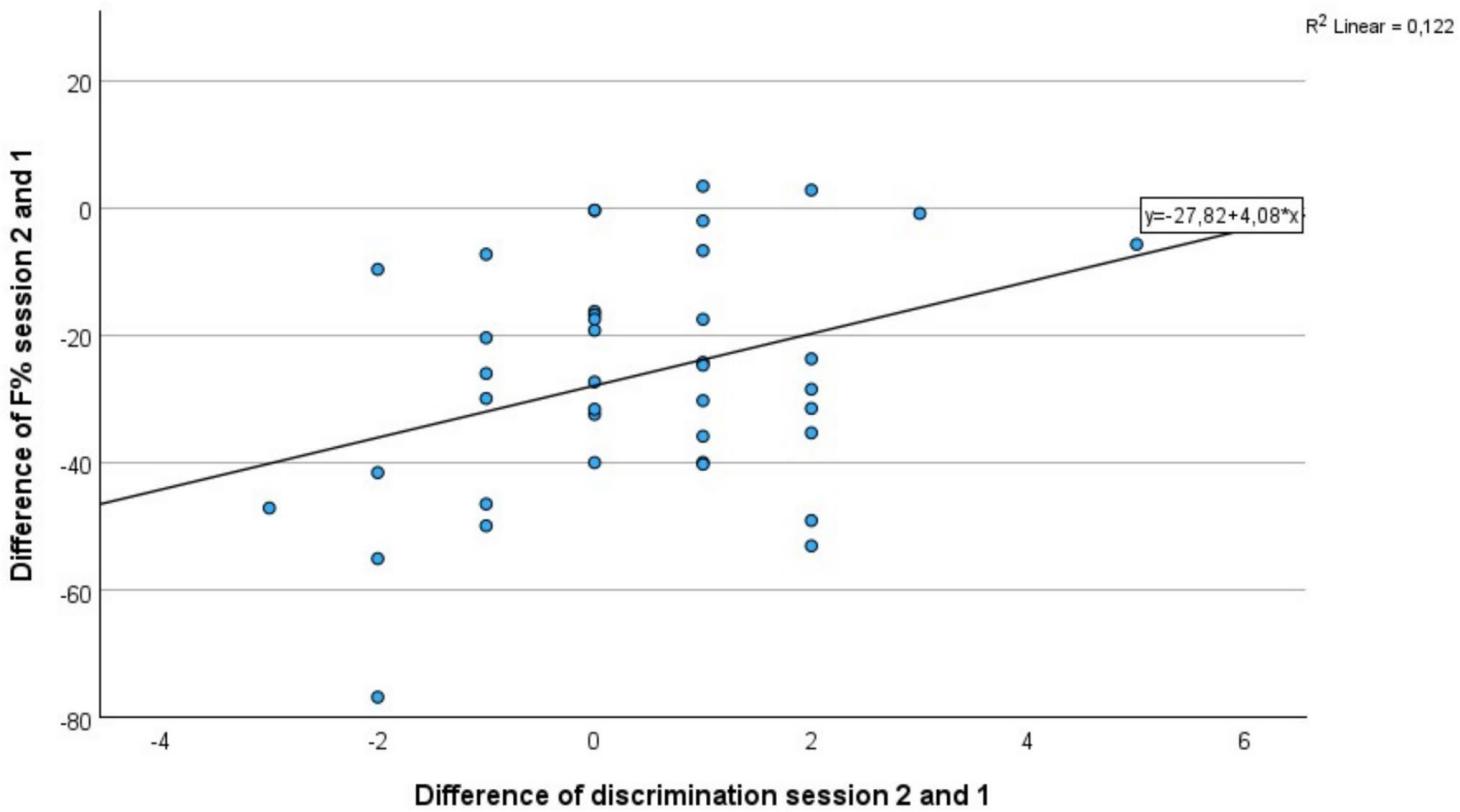
Scatter plot of difference between error rates between session 2 and 1 and difference of discrimination between session 2 and 1. (*n* = 40). Nearly all participants improved regarding the d2 test having lower error rates in session 2 compared to session 1 (see negative values for ∆ F%), whereas only a subset demonstrated improved olfactory function.

For individual values, there was a positive correlation between the d2 test of attention (concentration index, processing speed, accuracy) and olfactory discrimination, see Table 2 and Fig. 5. Participants with better olfactory discrimination showed better results for BZO (processed target objects) regarding sessions 1, 2, and the difference between both sessions (see Table 2). However, there was no correlation between the difference in discrimination (∆ discrimination sessions 1 and 2) and the difference in BZO (∆ BZO session 2/1) – addressing the possible effect of the TA-VNS – there was no correlation between the change in discrimination and the change in the d2 test of attention regarding BZO. There was a positive correlation between the difference in error rates and the difference in discrimination, indicating lower error rates went along with better olfactory discrimination improvements (*p* = 0.027, r = 0.349). While nearly all participants improved their error rates for the d2 test, only 47.5% improved the olfactory discrimination (25% without change of discrimination, 27.5% diminished olfactory discrimination). Furthermore, the concentration index (KL) of the first session was positively correlated with the change in olfactory discrimination between the two sessions. KL of the second session showed a positive correlation with olfactory discrimination in both sessions 1 and 2. Finally, the KL of the difference between the two sessions was positively correlated with olfactory discrimination in both the first and second sessions, see Table 2. When patients with OD were analyzed separately, positive correlations were observed between the difference of olfactory discrimination and BZO respectively KL for both sessions. In addition, olfactory discrimination in the second session showed a positive correlation with BZO in both sessions, see Table 3.

**Table 2:** Correlation of olfactory discrimination of session 1, 2 and the difference (∆) between session 2 and 1 regarding the d2 test of attention (BZO, KL, and F% of session 1 and 2) showing the *p*-value 2-tailed using Pearson correlation (r) for healthy individuals and patients with OD (*n* = 40). (** indicates significant correlation at the 0.01 level (2-tailed, * indicates significant correlation at the 0.05 level (2-tailed)

			D2 test of attention								
			BZO session 1	KL session 1	F% session 1	BZO session 2	KL session 2	F% session 2	∆ BZO session 2/1	∆ KL session 2/1	∆ F% session 2/1
Olfactory discrimination	session 1	r	,680^**^	-,109	,436^**^	,740^**^	,355*	,300	,515**	,593**	-,480**
		p	** <,001**	,503	**,005**	** <,001**	**,025**	,060	** <,001**	** <,001**	**,002**
	session 2	r	,751^**^	,096	,265	,763^**^	,437**	,174	,429**	,457**	-,302
		p	** <,001**	,554	,099	** <,001**	**,005**	,283	**,006**	**,003**	,059
	∆ session 2/1	r	-,013	,323*	-,329^*^	-,094	,060	-,239	-,218	-,304	,349*
		p	,934	**,042**	**,038**	,563	,712	,137	,178	,056	**,027**

**Table 3:** Correlation of olfactory discrimination of session 1, 2 and the difference (∆) between session 2 and 1 regarding the d2 test of attention (BZO, KL, and F% of session 1 and 2) showing the *p*-value 2-tailed using Pearson correlation (r) for patients with OD (*n* = 10). (** indicates significant correlation at the 0.01 level (2-tailed, * indicates significant correlation at the 0.05 level (2-tailed)

			D2 test of attention								
			BZO session 1	KL session 1	F% session 1	BZO session 2	KL session 2	F% session 2	∆ BZO session 2/1	∆ KL session 2/1	∆ F% session 2/1
Olfactory discrimination	session 1	r	,061	-,094	,350	-,073	-,277	,346	-,379	-,263	-,288
		p	,868	,796	,322	,841	,438	,327	,280	,462	,419
	session 2	r	,641*	,516	-,081	,647*	,355	-,097	,022	-,062	,051
		p	**,046**	,127	,824	**,043**	,314	,791	,953	,866	,890
	∆ session 2/1	r	,720*	,688*	-,364	,828**	,636*	-,380	,316	,128	,281
		p	**,019**	**,028**	,301	**,003**	**,048**	,279	,374	,725	,432

### Comparison of different stimulation sites in healthy controls

Comparing the different stimulation sites (TA-VNS, placebo (control condition), transcutaneous stimulation), there were no differences in olfactory threshold (*p* = 0.50), discrimination (*p* = 0.82), lateralization (*p* = 0.38), pleasantness ratings (*p* = 0.39–0.53) and the d2 test of attention (BZO: *p* = 0.78, F%: *p* = 0.87). For the intensity ratings for PEA, there was a trend for the stimulation site (F(2,58) = 3.16, *p* = 0.050), showing slightly lower intensity ratings for PEA after the stimulation following TA-VNS compared to the transcutaneous stimulation (*p* = 0.077, M_Diff_ = 0.63, 95%-CI[0.05, 1.31]). However, there were no main effects on the odors of fish (*p* = 0.87) and eucalyptol (*p* = 0.15).

## Discussion

In this study in 30 healthy participants and 10 patients with post-COVID-19 OD, the following main results emerged. First, olfactory discrimination improved for patients using TA-VNS as opposed to olfactory threshold. Second, odor intensity ratings increased for fish in patients and decreased for eucalyptol and PEA in healthy individuals following TA-VNS. Further, the patient’s pleasantness ratings for eucalyptol increased following TA-VNS. Third, for normosmic individuals, there were no changes for olfactory discrimination, identification, pleasantness ratings, and attention tasks regarding the three different stimulation sites. Fourth, regarding the d2 attention task both patients and healthy controls improved using VNS. Moreover, there was a positive correlation between olfactory discrimination and the d2 test of attention (BZO) for sessions 1 and 2.

In our study, olfactory discrimination improved in patients with OD using VNS whereas no such effect was observed for the olfactory threshold. These findings are in line with Maharjan et al., showing an improvement in suprathreshold olfactory function following high-frequency VNS using a similar paradigm [26]. However, in the study of Maharjan et al., vanillin was used in different concentrations, while our study tested the olfactory discrimination for different odors [32]. The persistence of the improvement of olfactory discrimination following VNS, independent from the use of different tests, emphasizes the effect of VNS on suprathreshold olfactory function. Our results were in contrast to other studies, that found no improvement in olfactory function following VNS [27–29]; however, those studies assessed olfactory function using the identification test of the Sniffin` Sticks [32].

There were no effects regarding the olfactory threshold neither for the comparison of different stimulation modalities in healthy subjects (TA-VNS, transcutaneous, placebo (control condition)) nor patients with OD following TA-VNS. Olfactory discrimination and identification require more complex cognitive abilities while olfactory threshold is more dependent on peripheral structures like the olfactory epithelium and cortical structures involved in low-level perceptual function [39]. A study on long-COVID patients demonstrated a mild to moderate positive effect on cognitive function, specifically in reducing mental fatigue symptoms, following at-home, self-administered low-frequency TA-VNS, though no assessment of olfactory function was conducted [40]. However, low-frequency stimulation, compared to high-frequency stimulation, did not result in measurable changes in olfactory perception [26].

Several cortical structures are actively involved in the processing of information from both olfaction and vagal nerve. The auricular branch of the vagus nerve (ABVN) transmits signals to the nucleus of the solitary tract (NTS). The NTS projects to several structures like the locus coeruleus (LC), which is an important noradrenergic brain structure. Thus, both the LC and the NTS are important targets of TA-VNS. Further, the NTS projects to the dorsal raphe nucleus (DRN), the parabrachial nucleus (PBN), and forebrain limbic structures like the paraventricular nucleus of the hypothalamus, the stria terminalis, and the cingulate cortex. The PBN projects to forebrain structures including the insular cortex, the amygdala, the thalamus, and the hypothalamus [15, 16, 41]. In addition, the LC modulates noradrenergic pathways such as the amygdala, the hippocampus, and the prefrontal cortex. Some of these structures overlap with the olfactory pathway. Following stimulation of olfactory receptor neurons, which first converge in the OB, OB neurons project via the lateral olfactory tract to the primary olfactory cortex including the piriform cortex, amygdaloid cortical nuclei, and entorhinal cortex [42, 43]. Subsequently, secondary olfactory areas become involved like the hippocampus, parahippocampal gyrus, thalamus, hypothalamus, insula, and orbitofrontal cortex [44]. Additional areas involved in olfaction are the nucleus accumbens, cingulate cortex, and cerebellum [44–46]. The role of the thalamus in olfaction is debated. Unlike other senses, the olfactory pathway was initially thought to bypass the thalamus, instead projecting to limbic areas before reaching it [47]. However, it has been shown since then that the mediodorsal thalamus receives projections from the primary olfactory cortex and might participate in the olfactory processing. To sum up, the TA-VNS targets the auricular branch of the vagus nerve that transmits signals to intermediate structures such as the locus coeruleus and the nucleus of the solitary tract, which modulates brain areas involved in the olfactory processing such as the amygdala and secondary olfactory structures.

There is a close link between olfaction, gustation, and deglutition in terms of a protective function [48], which is seen at the physiological level and an overlap of cortical structures involved in the cortical swallowing network [49], where the vagal nerve has an important impact on the latter. It has been shown that after retronasal presentation of odors swallowing occurred significantly faster and took place more frequently compared with swallowing after orthonasal odor presentation [50]. The large number of jointly activated cortical areas of olfaction and the afferent vagal nerve system could explain the effect of improved olfaction as a result of VNS.

Nevertheless, the effect of the VNS on olfaction could not be conclusively clarified. The main limiting factors were the small number of patients with OD and the missing placebo stimulation (control condition) in patients with OD. In a follow-up study, the effect of VNS on patients with OD should be investigated including a placebo-controlled stimulation. Anecdotally, four patients reported during a follow-up appointment in the clinic that they experienced vastly improved olfactory function in the evening following stimulation. In future studies, it would be valuable to conduct additional measurements over time after stimulation to capture potential temporal effects. For example, such a temporal effect was observed for trigeminal stimulation, as an improvement in olfactory threshold 30 min after trigeminal stimulation using trigeminal nerve stimulation (TNS) or transcranial direct current stimulation, but not immediately after the stimulation [24]. Thus, stimulation of other nerves can also affect olfactory function. The auricle receives sensory innervation not only from the vagus nerve in the area of the concha but also from the cervical plexus, the glossopharyngeal nerve, and the trigeminal nerve [51].

The electrical waveform characteristics, such as intensity, shape, and frequency, as well as electrode-specific factors like material, size, and location, play an important role in the stimulation [12]. Maharjan et al. (2018) showed an improvement in olfactory function for the high frequencies while lacking for low frequencies [27]. In future studies, different parameters could be tested to analyze the results as a function of these parameters.

The tests used to evaluate the olfactory function appear to play a role in detecting the effects of VNS. In healthy participants, no improvement in olfactory discrimination was observed following VNS, which may be attributed to a ceiling effect. Healthy participants already had very high scores, and as the test is designed to differentiate between healthy and impaired patients, it was unable to detect changes in olfactory discrimination. This result appears to contrast with the findings of Maharjan et al., who demonstrated an improvement even in healthy participants using a suprathreshold test. However, the results were aggregated and included normosmic, hyposmic, and anosmic patients [26], which probably influenced their results. A potential improvement in healthy individuals, similar to Maharjan et al. might be observable with a larger sample size or by using a different method to test olfactory discrimination (e.g. an odor sorting task [52]). In addition, previous studies did not show any changes regarding the odor identification task [27–29].

Our study showed an improvement in attention using the d2 test for patients with OD and healthy subjects following VNS. This aligns with the study of Zheng et al., who found an improvement for Long COVID-19 patients of attention following VNS using the Flanker Inhibitory Control and Attention Test as well as an improvement for anxiety, depression, and sleep with benefits staying at a similar level for a one-month follow-up [29]. For healthy individuals, we showed a more pronounced improvement in attention following the TA-VNS compared to patients with OD. However, simply repeating the d2 test can lead to improved results [53], which healthy individuals repeated four times. This could also explain why no differences in attentional improvement were observed between the three conditions (TA-VNS, placebo stimulation (control condition) with electrodes placed at the TA-VNS, forearm) in healthy participants. A recent review on VNS showed an improvement in attention and working memory in patients with epilepsy or mood disorders. However, it concluded that there was insufficient evidence to establish VNS as an enhancer of attention and working memory in patients with neuropsychiatric disorders [54]. The present study showed that participants with higher scores for attention had better olfactory discrimination, however it was insistently for values of processing speed, precision and concentration index. Considering the difference between the two measurement times, which addresses the possible effect of the TA-VNS, correlations between changes in olfactory discrimination and the d2 attention test were observed for some values across all participants, but not consistently. In healthy controls, a ceiling effect in olfactory discrimination likely limited interpretability. In patients with OD, changes in olfactory discrimination correlated for instance with the concentration indices of sessions 1 and 2, but not with the change in the concentration index. However, the small patient sample limits the statistical power of these findings. To conclude, for individual values, concentration index, processing speed and accuracy showed a correlation with olfactory discrimination to some extent, which aligns with the existing literature. Nevertheless, the improvement in discrimination following TA-VNS is unlikely to be solely explained by increased attention, given the inconsistency of the effect. Additional studies involving larger cohorts are needed to further examine this effect.

The present results highlight the possible improvement of olfactory discrimination in patients following TA-VNS which should be re-examined in further studies.

## Conclusion

In post-COVID-19 patients with OD, TA-VNS is associated with an improvement in odor discrimination, an increase in odor intensity for fish, and higher pleasantness of Eucalyptol. These results seem to underline the functional connection of the olfactory system with the afferent vagus network. However, a ceiling effect for olfactory discrimination did not allow to observe a possible improvement in normosmic individuals.

## Supplementary Information

Below is the link to the electronic supplementary material.

Supplementary file1 (DOCX 42 KB)
