# Supplementary material for: Improved olfactory function following high-frequency transcutaneous auricular vagal nerve stimulation in post-COVID-19 patients with olfactory dysfunction
Source: Eur Arch Otorhinolaryngol. 2025 Oct 18;282(12):6269–80. doi: 10.1007/s00405-025-09701-5 (PMC12680794; doi:10.1007/s00405-025-09701-5)
Supplement: Supplementary file 1 — Supplementary file1 (DOCX 42 KB) [file 405_2025_9701_MOESM1_ESM.docx]

# Supplementary

Table 4: Participants age in years by etiology and gender

| etiology | olfactory status | sex | N | Mean | Median | SD | Minimum | Maximum |
| --- | --- | --- | --- | --- | --- | --- | --- | --- |
| post viral | anosmic | male | 0 | NaN | NaN | NaN | NaN | NaN |
|  |  | female | 2 | 54.0 | 54.0 | 1.4 | 53.0 | 55.0 |
|  | hyposmic | male | 1 | 61.0 | 61.0 | NaN | 61.0 | 61.0 |
|  |  | female | 7 | 49.9 | 58.0 | 14.1 | 23.0 | 61.0 |
| healthy | normosmic | male | 15 | 24.3 | 24.0 | 1.5 | 23.0 | 29.0 |
|  |  | female | 15 | 36.5 | 26.0 | 2.9 | 23.0 | 32.0 |

Table 5: Participants TDI by gender.

| etiology | sex | N | Mean | Median | SD | Minimum | Maximum |
| --- | --- | --- | --- | --- | --- | --- | --- |
| post viral | male | 1 | 23.0 | 23.0 | NaN | 23.0 | 23.0 |
|  | female | 9 | 20.3 | 19.0 | 4.2 | 14.0 | 26.5 |
| healthy | male | 15 | 39.4 | 38.3 | 4.0 | 32.5 | 45.5 |
|  | female | 15 | 38.5 | 38.0 | 4.0 | 32.3 | 47.0 |

Table 6: Participants olfactory discrimination and threshold by categorization of olfactory function.

| etiology | N | Olfactory discrimination | | | | | Olfactory threshold | | | | |
| --- | --- | --- | --- | --- | --- | --- | --- | --- | --- | --- | --- |
|  |  | Mean | Median | SD | Min | Max | Mean | Median | SD | Min | Max |
| post viral | 10 | 9.3 | 9.0 | 1.3 | 7.0 | 11.0 | 2.6 | 1.3 | 2.1 | 1 | 6.25 |
| healthy | 30 | 14.5 | 15.0 | 1.3 | 12 | 16 | 10.3 | 10.3 | 3.0 | 4.5 | 15.8 |

**Comparison of healthy and patients with OD**

Healthy controls had higher threshold scores compared to patients (F(1,38)=67.60, p<0.001, MDiff = 7.78, 95%-CI[5.86, 9.69]). Healthy individuals outperformed patients with OD regarding discrimination scores (F(1, 38) = 108.29, *p* < 0.001, MDiff = 4.62, 95%-CI[3.72, 5.52]). Further, healthy controls showed a better lateralization ability than patients with OD (F(1,38)=5.61, p=0.023, MDiff=1.97, 95%-CI[0.29, 3.65]).

Healthy individuals rated the odors more intense than patients (PEA: F(1,38)=22.64p<0.001, MDiff=2.80, 95%-CI[1.61, 3.99], Eucalyptol: F(1,38)=8.23, p=0.007, MDiff=1.85, 95%-CI[0.54, 3.16]; fish: F(1,38)=23.64, p<0.001, MDiff=2.90, 95%-CI[1.69, 4.11]). Further, healthy controls rated the odors PEA and Eucalyptol more pleasant than patients (PEA: F(1,38)=12.92, p<0.001, MDiff=2.38, 95%-CI[1.04, 3.73], Eucalyptol: F(1,38)=35.04, p<0.001, MDiff=3.17, 95%-CI[2.08, 4.25]; but not fish: p=0.16).

Healthy individuals outperformed patients regarding the d2 test of attention (BZO: F(1,38)=40.07, p<0.001, MDiff=76.27, 95%-CI[51.88, 100.66]; F%: F(1,38)=19.57, p<0.001, MDiff = 35.50, 95%-CI[19.25, 51.75]).

Table 7 Participants results for the d2 test.

| etiology  N | | Session1 | | | | | | Session 2 | | | | | |
| --- | --- | --- | --- | --- | --- | --- | --- | --- | --- | --- | --- | --- | --- |
|  |  | BZO | | KL | | F% | | BZO | | KL | | F% | |
|  |  | Mean | SD | Mean | SD | Mean | SD | Mean | SD | Mean | SD | Mean | SD |
| post viral | 10 | 129.5 | 38.7 | 111.5 | 46.7 | 16.7 | 19.8 | 147.3 | 38.9 | 121.7 | 59.9 | 10.9 | 10.9 |
| healthy | 30 | 191.4 | 30.6 | 77.1 | 65.4 | 65.9 | 29.1 | 238.2 | 33.9 | 166.7 | 69.1 | 32.7 | 19.9 |
